# Use of platelet lysate from estrus heifer for *in vitro* embryo production in dairy cows

**DOI:** 10.3389/fvets.2026.1940687

**Published:** 2026-09-07

**Authors:** Silviu-Ionuț Borş, Vasile Vintilă, Alina Borş, Amalia-Ioana Hârbu, Viorel-Cezar Floriștean

**Affiliations:** 1 Research and Development Station for Cattle Breeding Dancu, Iaşi, Romania; 2 “Ion Ionescu de la Brad” Iasi University of Life Sciences, Iasi, Romania

**Keywords:** dairy heifer, embryos, hatched blastocysts, *in vitro* culture, platelet lysate

## Abstract

**Introduction:**

Enhancing the efficiency of bovine *in vitro* embryo production (IVEP) requires continuous optimization of culture microenvironments. Fetal bovine serum (FBS) remains the standard biological supplement but introduces issues related to batch variability and compromised embryonic ultrastructure. Consequently, identifying alternative, standardized biological supplements is essential to enhance developmental competence.

**Methods:**

This study evaluated the effects of supplementing *in vitro* culture (IVC) medium with 5% platelet lysate (PL), derived from the platelet-rich plasma (PRP) of a single heifer selected during spontaneous estrus, on bovine blastocyst development and hatching rates. A total of 78 ovaries from 39 high-yielding Holstein cows were opportunistically collected from a local abattoir across 5 independent IVEP sessions. To eliminate donor-specific confounding bias, retrieved cumulus-oocyte complexes (COCs) were completely pooled within each session prior to morphological grading and random distribution into either the PL-supplemented group or the IVC Control group (C-group). Across the 5 replicates, a cumulative total of 632 follicles were aspirated (mean: 126.4 ± 8.14 per session), yielding 550 recovered COCs (mean: 110 ± 6.91 per session) and achieving a mean recovery rate of 86.53 ± 3.56%.

**Results:**

Using the independent session as the experimental unit (*n* = 5; paired analysis), 5% PL supplementation significantly increased the proportion of hatched blastocysts on Day 8 of culture (40.33 ± 3.54% vs. 16.71 ± 1.27% in the C-group; *p* = 0.007), indicating accelerated hatching kinetics. The cumulative blastocyst yield on Day 8 (expanded plus hatched blastocysts relative to the allocated COCs) was numerically higher in the PL-group (42.75 ± 4.00% vs. 28.28 ± 1.97%) but only approached statistical significance (*p* = 0.06). Cleavage and morula formation rates, together with all expanded-blastocyst rates, were comparable between the two groups.

**Discussion:**

Supplementing bovine embryo culture medium with 5% PL derived from an estrus heifer significantly increases blastocyst hatching on Day 8, with a positive but non-significant trend for the overall blastocyst yield. These findings highlight the efficacy of estrus-specific platelet-derived factors as a viable alternative to standard biological supplements for optimizing IVEP outcomes in cattle.

## 1 Introduction

*In vitro* embryo production (IVEP) in cattle is a cornerstone of advanced reproductive biotechnologies, relying heavily on the optimization of *in vitro* culture (IVC) systems to mimic the physiological environment of the oviduct and uterus (1–3). To achieve acceptable blastocyst development rates, IVC media require supplementation with proteins, growth factors, and signaling molecules (4, 5). Historically, fetal bovine serum (FBS) has been the most widely used biological supplement in bovine IVC. However, FBS supplementation presents significant limitations, including high batch-to-batch variability, lack of standardization, and documented adverse effects on embryo ultrastructure, premature compaction, altered gene expression, and compromised cryotolerance (6, 7). Consequently, there is a critical need to identify defined or alternative biological supplements that can enhance embryo developmental competence and quality without the detrimental side effects of FBS. Recent scientific strategies have focused on shifting away from FBS in embryo culture due to compounding ethical, safety, and reproducibility challenges. Contemporary *in vitro* production research explores chemically defined components and advanced blood-derived alternatives to mimic the physiological environment and stabilize embryonic development (8, 9).

To improve IVC performance, researchers investigate various blood-derived products such as platelet-rich plasma (PRP) and platelet lysate (PL), which must be differentiated based on their biological composition and preparation. These distinct biological supplements provide unique growth factors and metabolic drivers that support cellular proliferation and embryo development (10). Platelet-rich plasma is an autologous or allogeneic concentration of platelets in a small volume of plasma, obtained through specific centrifugation protocols (11). Platelet lysate, on the other hand, is generated by disrupting the platelet membranes—often through freeze–thaw cycles—to release a highly concentrated cocktail of growth factors, cytokines, and chemokines originally encapsulated within the platelet α-granules (12, 13). Distinct from platelet derivatives, estrus cow serum is the liquid portion of coagulated whole blood collected specifically during the estrus phase; it is naturally rich in gonadotropins, tightly regulated levels of estradiol, and systemic metabolic factors that stimulate cellular proliferation and survival (14). While PRP and PL provide potent angiogenic, mitotic, and antimicrobial properties via platelet-released proteins (13, 15), estrus cow serum provides a distinct, hormone-rich matrix that has shown superior performance compared to standard FBS in various bovine developmental assays (14).

The therapeutic and culturing benefits of platelet-derived factors are well-documented, showing a threshold effect where lower concentrations (e.g., 5%) optimize cellular responses, while higher concentrations (e.g., 10%) may inhibit development (16, 17). Recently, the application of platelet derivatives has been extended to bovine embryology. Specifically, Hassaneen et al. (18) demonstrated that the inclusion of PL during IVEP can support bovine embryo development *in vitro* (18). However, the specific physiological state of the blood donor remains an unexplored variable that could critically influence the efficacy of the PL. The systemic hormonal and biochemical fluctuations characteristic of the bovine estrus cycle—particularly elevated estrogen levels—significantly alter blood composition and platelet activity, potentially enriching the growth factor profile of the PL.

To date, the specific impact of utilizing PL derived from the PRP of heifers strictly selected during the estrus phase remains uncharacterized. This study aims to address this gap by investigating the potential synergistic benefits of incorporating a 5% PL, obtained specifically from an estrus heifer, into bovine IVC media. We hypothesize that combining the platelet-derived growth factor cocktail with the specific physiological background of estrus donors will enhance blastocyst development and hatching rates compared to standard protocols.

## 2 Materials and methods

### 2.1 Study design and session allocation

The study was prospectively structured across 5 independent replications (IVEP sessions), along with IVF media (IVF Bioscience, Bickland Industrial Park, Falmouth, United Kingdom), as indicated on the vials: OPU medium, BO-HEPES-IVM medium, BO-IVF fertilization, Oocyte and Embryo Wash Medium, Oil for Medium Overlay, BO-IVC embryo culture medium, and BO-SemenPrep medium. All commercial IVF media (IVF Bioscience, Bickland Industrial Park, Falmouth, United Kingdom) were maintained in unopened vials at 0–4 °C, protected from light, and equilibrated prior to use according to the manufacturer’s instructions. The pH levels remained stable between 7.2 and 7.4 throughout the experimental procedures, aligning with established benchmarks (19).

Because ovaries were obtained opportunistically from a local abattoir according to the daily slaughter schedule, the total number of donor cows available varied between collection days. To limit donor-specific bias and avoid confounding initial oocyte quality with the culture treatment, all cumulus-oocyte complexes (COCs) retrieved within each independent session were pooled together. Only after this pooling and subsequent morphological grading were the COCs randomly distributed into either the Control group (C-group) or the Platelet Lysate group (PL-group). Consequently, the experimental unit in this study was defined as the independent IVEP session (replicate; *n* = 5). Retrospective tracking of the abattoir batches logged a total of 78 ovaries harvested across the 5 independent replicates, reflecting the opportunistic slaughter distribution on the collection days. Because all retrieved COCs were pooled and randomized within each session prior to group allocation, this variation in the number of donor animals related to baseline abattoir logistics rather than to the culture treatment. Culturing the two treatments within the same session (session-blocked design) further reduced the influence of between-batch variability on the treatment comparison.

The final sample size was justified based on established parameters in bovine IVEP literature, where a cumulative pool of 200–300 cultured oocytes per experimental group distributed across 4–5 independent replications is mathematically required to achieve a statistical power of ≥ 80% (with α = 0.05) for detecting a 10–15% difference in blastocyst development rates (16, 17). With a cumulative recovery of 550 COCs across 5 independent replicates (238 allocated to the PL-group and 312 to the C-group), the sample size in the present study provided sufficient statistical power and degrees of freedom to rigorously validate the results.

### 2.2 PL derived from PRP of estrus heifer

PRP and subsequent PL were derived from a single (*n* = 1) 14-month-old, healthy, non-pregnant Holstein-Friesian heifer. The donor animal was selected based on a flawless reproductive history and confirmed health status via rigorous clinical examination, displaying no signs of systemic or local pathologies. Blood collection was strictly timed to coincide with a spontaneous estrus event. The onset of estrus was monitored using behavioral indicators (standing estrus, increased alertness, and mounting behavior) and confirmed clinically via transrectal palpation demonstrating elevated uterine tone. Additionally, the presence of abundant, clear, cohesive, and transparent estrus mucus, discharged upon transrectal uterine massage, served as the definitive diagnostic criterion. After 12 h after the end of the estrus, the heifer was artificially inseminated and diagnosed as pregnant on day 30 after insemination.

A total of 20 mL of peripheral whole blood was aseptically collected from the coccygeal vein into 2 sterile 13 mL vacuum tubes, each one preloaded with 1 mL sodium citrate anticoagulant and 1.5 mL thixotropic separator gel (Rejuvinova^®^, Jiangsu Yuli Medical Instrument, China). Immediately after collection, the tubes were gently inverted 5–10 times to ensure proper mixing and allowed to rest at room temperature for 5–10 min to promote platelet stabilization. Centrifugation was performed at 500 × g for 10 min at room temperature. Following centrifugation, the PRP fraction positioned above the separator gel barrier was carefully isolated. The PRP layer was aspirated through the tube stopper using a sterile syringe fitted with an 18G needle (Prima Medical Ltd., United Kingdom), ensuring the gel barrier remained completely undisturbed to prevent red blood cell or leukocyte contamination.

To convert the isolated PRP into PL via mechanical platelet activation, the samples were subjected to three rapid freeze–thaw cycles (−80 °C to 37 °C) to induce complete structural lysis and release of encapsulated growth factors (20). Following the final thawing cycle, the lysed suspension was centrifuged at 700 × g for 10 min to precipitate cellular fragments and debris. The resulting growth-factor-enriched supernatant (now designated as PL) was harvested and filtered through a 0.22 μm membrane filter (Millipore, Bedford, MA, United States) to ensure sterility. The final PL batch was aliquoted into sterile 1.5 mL Eppendorf tubes (Sigma-Aldrich, United States) in 0.25 mL working volumes according to daily culture requirements and stored at −80 °C. This single, uniform maternal batch of PL precluded the need for multi-donor pooling and was used consistently across all five independent IVEP session replicates to maintain experimental homogeneity.

### 2.3 Ovary collection and oocyte recovery

The study was conducted using ovaries collected from a local abattoir. A total of 78 ovaries were obtained from Holstein Friesian cows raised on a high-yielding dairy farm with an average milk yield of approximately 50 kg/day. The donor cows belonged to a culled group, with a milk production on the day of ovary harvest of 30 ± 9.14 kg/cow/day and an average parity of 2.8 ± 0.9. Ovaries were recovered within a maximum of 10 min post-slaughter to ensure maximum viability and were placed in a thermal flask containing 0.9% saline solution (Braun, Romania) supplemented with 50 μg/mL of gentamicin (Pasteur, Romania) to prevent bacterial contamination. The transport medium was maintained at a temperature between 28 and 35 °C, which was necessary to preserve ovarian tissue integrity. The collected ovaries were transported to the IVF laboratory in approximately 2 h.

Before follicle aspiration, the ovaries were carefully washed three times in sterile 0.9% saline solution maintained at a temperature of approximately 35 °C. To avoid aspiration of residual saline solution during the COCs collection procedure, each ovary was gently dried using sterile paper towels.

For follicle aspiration, an 18G sterile needle (Prima Medical Ltd., United Kingdom) attached to a 10 mL syringe (Jiangsu Kanghua Medical Equipment, China) was used. The follicular fluid, containing immature COCs, was collected into sterile Falcon tubes (Sigma-Aldrich, United States) containing sterile OPU medium.

Across the 5 independent collection sessions, a cumulative total of 632 follicles were aspirated, yielding an average of 126.4 ± 8.14 follicles per session. This procedure resulted in the recovery of 550 COCs in total (mean: 110 ± 6.91 COCs per session), demonstrating an overall mean oocyte recovery rate of 86.53 ± 3.56% per individual replication.

### 2.4 Cumulus-oocyte complex evaluation and *in vitro* maturation

Following follicular aspiration, the collected follicular fluid was transferred into a pre-warmed Petri dish maintained at a temperature of 37 °C and containing OPU medium. Using a stereomicroscope (Olympus SZ-51, Olympus, Hachioji, Tokyo), all COCs were carefully recovered with a 10 μL pipette (BRAND Transferpette^®^, Germany) fitted with sterile filter tips (BRAND, Germany).

The recovered COCs were washed three times in a specialized Oocyte and Embryo Wash Medium to remove any residual follicular and OPU fluid. Subsequently, each COC was classified into one of four quality classes (A–D) based on specific criteria related to the morphology of the cumulus cell layers and the overall aspect of the ooplasm. The classification was determined by assessing the number and compactness of cumulus cell layers, along with visual characteristics of the ooplasm such as granularity, coloration, and density.

Class A COCs exhibited several compact and dense layers of cumulus cells, indicating a strong supporting structure around the oocyte, which is essential for optimal maturation and developmental potential. Class B COCs also presented compact cumulus cell layers, although with slightly reduced density compared with Class A, suggesting a lower supportive quality, yet still maintaining some level of cohesion around the oocyte. Class C COCs showed partial denudation of cumulus cell layers, characterized by thin or incomplete layers of cumulus cells that offered minimal support, potentially compromising the oocyte’s development. Finally, Class D COCs were largely denuded, presenting either completely absent or very few remaining cumulus cells, which may severely hinder the ability of the oocyte to undergo proper maturation and fertilization (21).

Following morphological evaluation, COCs from all four quality classes (A–D) were allocated into the maturation system. The inclusive use of lower-grade oocytes (Classes C and D) was designed to evaluate the efficacy of alternative media supplements on unselected abattoir-derived COCs, closely mimicking practical field conditions. Because all COCs harvested within the same session were fully pooled and thoroughly mixed prior to allocation, the allocation process was entirely randomized.

Prior to use, the BO-HEPES-IVM medium was preheated in the incubator for exactly 1 h to reach 38.8 °C, avoiding extended gas pre-equilibration. Following morphological evaluation and sorting, cohorts of approximately 50 COCs were introduced into 1 mL of the preheated medium and subsequently incubated for 24 h at 38.8 °C in a humidified atmosphere containing 5.5% CO_2_, strictly adhering to the manufacturer’s validated technical protocol (IVF Bioscience).

The selection of the BO-HEPES-IVM formulation was strategically based on its dual-buffering matrix (sodium bicarbonate combined with HEPES), which safeguards the oocytes against damaging pH shifts during necessary atmospheric exposure on the laboratory bench during sorting and washing steps. Furthermore, the 5.5% CO_2_ atmospheric concentration was strictly applied in accordance with the manufacturer’s technical specifications for IVF Bioscience media, guaranteeing optimal gas- liquid equilibration and maintaining the target physiological pH of 7.2–7.4 throughout the IVM phase.

### 2.5 *In vitro* fertilization of oocytes and presumptive embryo culture

After the maturation process, oocytes were washed at least three times using BO-IVF medium to remove residual factors that could compromise fertilization. The washed oocytes were subsequently placed in 90 μL microdrops of BO-IVF fertilization medium and carefully overlaid with mineral oil to prevent evaporation and maintain osmotic balance. Prior to use, the microdrops of BO-IVF medium were equilibrated for at least 2 h at a precise temperature of 38.8 °C in a standard CO_2_ incubator, ensuring an optimal 5.5% CO_2_ concentration in a humidified atmosphere.

For the IVF procedures, frozen–thawed spermatozoa from a sire with proven fertility were used to ensure high-quality gametes. The semen straws were thawed at 37 °C for 30 s and subsequently subjected to two rounds of centrifugation (5 min at 328 × g) in preheated (37 °C) 4 mL and 2 mL of BO-SemenPrep medium for semen preparation. This centrifugation process was conducted under ambient air conditions and was essential for selecting motile spermatozoa while removing cryoprotectants and cellular debris. Following centrifugation, the excess BO-SemenPrep medium was carefully aspirated, and the resulting semen pellet was resuspended in approximately 200 μL of BO-SemenPrep medium. An aliquot containing approximately 1 × 10^6^ sperm/mL (5–8 μL of the resuspended sperm pellet) was added to the 90 μL BO-IVF microdrops, followed by coincubation of the gametes for 18–22 h at 38.8 °C in a humidified incubator under a controlled atmosphere containing 5.5% CO_2_ in ambient air.

The introduction of this minimal volume of BO-SemenPrep into the BO-IVF microdrop strictly followed the manufacturer’s co-validated standard protocol (IVF Bioscience). Due to the low volume ratio (5.2–8.1% of the total volume), this minor carryover did not alter the final pH, osmolarity, or bicarbonate buffering capacity of the fertilization microenvironment, maintaining the target pH of 7.2–7.4 throughout the co-incubation period.

Following fertilization, for the cultivation of presumptive zygotes, the surrounding cumulus cells were mechanically removed, and the presumptive zygotes were washed three times in a dedicated Oocyte and Embryo Wash Medium. This washing procedure ensured the removal of excess cumulus cells and spermatozoa debris that could interfere with subsequent development. The washed presumptive zygotes were cultured in microdrops of BO-IVC embryo culture medium, approximately 25 presumptive zygotes/microdrop. For the C-group, 100 μL microdrops were used, while in the PL-group, 95 μL microdrops of the same medium were supplemented with 5 μL of prepared PL derived from an estrus heifer. These culture microdrops were placed under a protective layer of mineral oil for medium overlay. All culture procedures were performed under strictly controlled conditions, following the manufacturer’s recommendations (38.8 °C in a standard CO_2_ incubator with a humidified atmosphere containing 5.5% CO_2_ in ambient air) to ensure optimal performance. Prior to use, the prepared culture microdrops were equilibrated for a minimum of 2 h at 38.8 °C in the CO_2_ incubator under a humidified atmosphere containing 5.5% CO_2_.

Throughout all IVEP procedures—including *in vitro* maturation, fertilization, and embryo culture—35/10 mm sterile cell culture dishes (Cellstar^®^ TC, Greiner Bio-One GmbH, Frickenhausen, Germany) were used to maintain a stable environment. The outcomes of the IVEP procedures were evaluated on Days 7 and 8, with the day of fertilization designated as Day 0.

### 2.6 Statistical analysis

In each of the five independent IVEP sessions (replicates; *n* = 5), all cumulus–oocyte complexes (COCs) recovered from the pooled follicular aspirate were graded and then allocated between the Control (C-group) and platelet lysate (PL-group) groups prior to maturation. The independent session was therefore defined as the experimental unit. For each endpoint, a proportion (%) was calculated within every session relative to the appropriate denominator: oocyte-class proportions and the cleavage (2-cell) rate relative to the COCs allocated to each group; the morula rate relative to 2-cell embryos; the expanded- and hatched-blastocyst rates relative to morulae; and the cumulative blastocyst yield (expanded plus hatched blastocysts on Day 8) relative to the allocated COCs. This produced five per-session proportions per group for each endpoint. Because both groups originated from the same pooled COCs within each session, group means were compared with a paired two-tailed Student’s *t*-test (n = 5 sessions). Data are presented as the mean of the per-session proportions ± standard error of the mean (SEM). Differences were considered statistically significant at *p* ≤ 0.05. Statistical analyses were performed using GraphPad Prism v. 11.0.2.

## 3 Results

**Figure 1:**
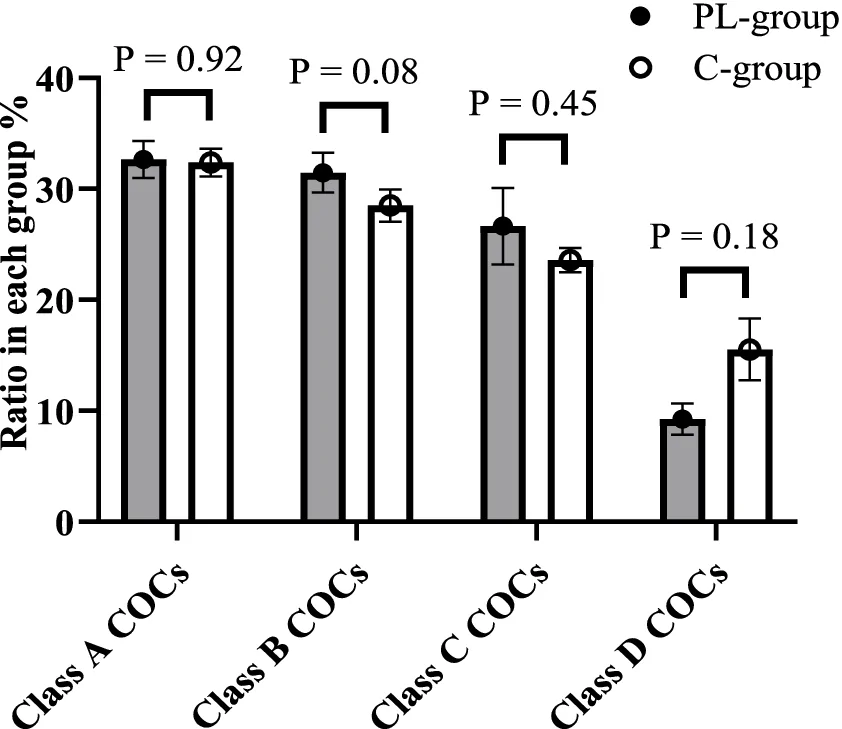
Comparison of each COCs class between PL-group vs. C-group. Values are presented as mean ± standard error of the mean.

Following the session-based pooling and subsequent allocation of cumulus–oocyte complexes (COCs), the two groups were comparable at baseline. The proportions of Class A, Class B, Class C and Class D oocytes did not differ significantly between the PL-group and the C-group (*p* > 0.05) (Table 1 and Figure 1). Although the PL group had a slightly higher proportion of Class B oocytes compared to the C-group (*p* = 0.08), this difference represented only a non-significant trend. This indicates that group allocation from the common pool did not introduce a significant baseline imbalance in oocyte morphology.

**Table 1:** Comparison of baseline oocyte quality and embryo developmental rates between the PL-group and C-group.

Items	PL-group	C-group	*p*-value	Total
Session-level recruitment (pooled COCs, then allocated)				
COCs recovered and allocated (n)	238	312	–	550
COCs quality—n (mean % of allocated COCs ± SEM)				
Class A COCs	78 (32.64 ± 1.67)	101 (32.38 ± 1.25)	0.92	179
Class B COCs	75 (31.46 ± 1.79)	88 (28.50 ± 1.45)	0.08	163
Class C COCs	62 (26.64 ± 3.45)	75 (23.58 ± 1.10)	0.45	137
Class D COCs	23 (9.26 ± 1.40)	48 (15.53 ± 2.79)	0.18	71
Embryo development—n (mean % ± SEM per session)				
2-cell embryos *(% of allocated COCs)*	164 (70.36 ± 3.73)	197 (64.85 ± 4.15)	0.46	361
Morulae *(% of 2-cell)*	109 (66.61 ± 0.80)	146 (75.83 ± 3.25)	0.07	255
Expanded blastocysts, Day 7 *(% of morulae)*	45 (40.17 ± 4.63)	43 (29.22 ± 3.61)	0.16	88
Expanded blastocysts, Day 8 *(% of morulae)*	55 (50.17 ± 2.73)	62 (41.77 ± 5.05)	0.11	117
Hatched blastocysts, Day 7 *(% of morulae)*	7 (6.61 ± 1.36)	7 (4.70 ± 0.53)	0.25	14
Hatched blastocysts, Day 8 *(% of morulae)*	43 (40.33 ± 3.54)	24 (16.71 ± 1.27)	0.007	67
Cumulative blastocyst yield, Day 8 *(% of allocated COCs)*	98 (42.75 ± 4.00)	86 (28.28 ± 1.97)	0.06	184

For each cell, the count is the cumulative total across the five independent sessions and the value in parentheses is the mean of the per-session proportions (%) ± SEM. *p*-values are from paired two-tailed Student’s *t*-tests comparing the five per-session proportions of the PL- and C-groups, with the independent session as the experimental unit (*n* = 5). *p* ≤ 0.05 was considered significant.

**Figure 2:**
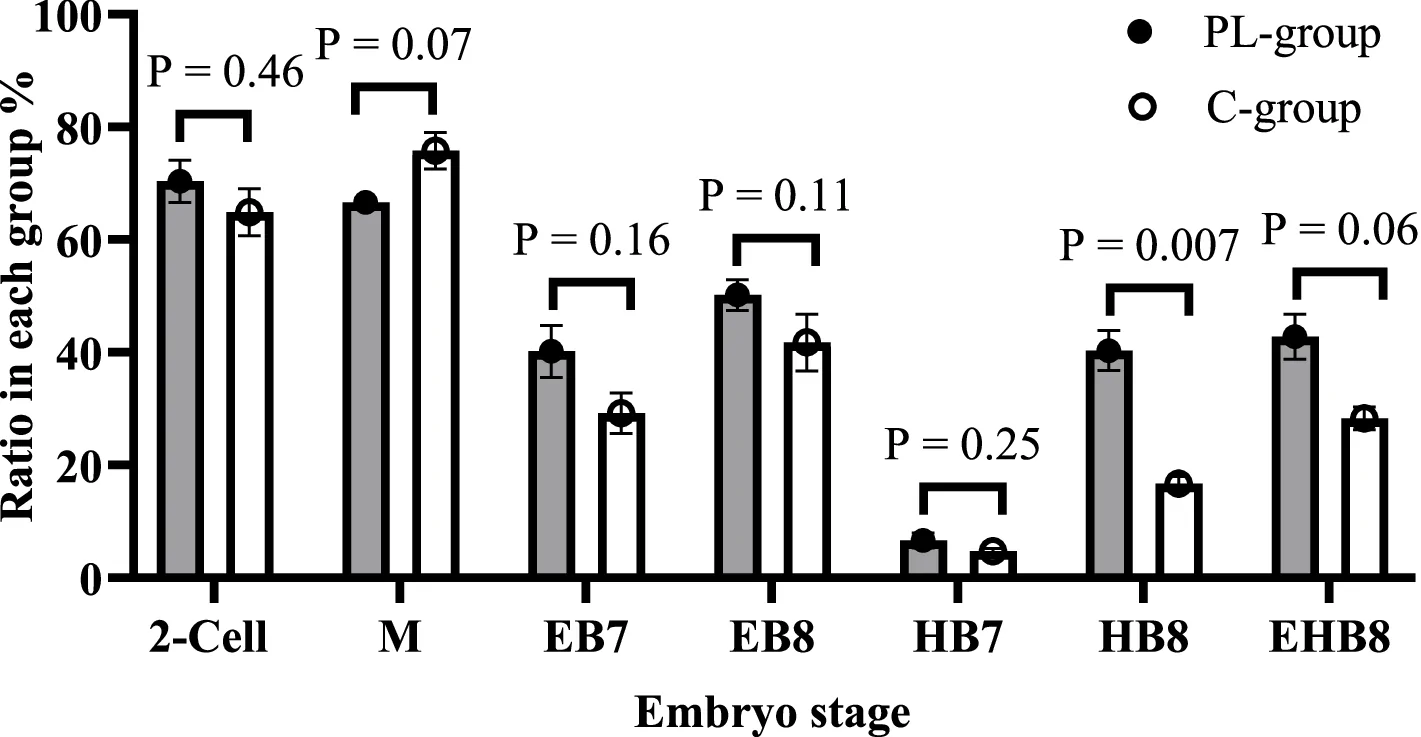
Comparison of developmental rates for each embryo stage between the PL-group and the C-group. 2-Cell, 2-cell stage embryo rate from cultured oocytes; M, morula stage rate from 2-cell stage embryos; EB7, Day 7 expanded blastocyst stage rate from morula stage embryos; EB8, Day 8 expanded blastocyst stage rate from morula stage embryos; HB7, Day 7 hatched blastocysts rate from morula stage embryos; HB8, Day 8 hatched blastocyst rate from morula stage embryos; EHB8, Day 8 cumulative blastocyst rate (both expanded and hatched) from recovered COCs. Values are presented as mean ± standard error of the mean.

At the first developmental evaluation, the cleavage (2-cell) rate calculated from the allocated COCs was similar between the two groups (*p* > 0.05) (Table 1), indicating comparable early mitotic progression. The subsequent morula rate derived from 2-cell embryos was numerically higher in the C-group than in the PL-group (75.83 ± 3.25% vs. 66.61 ± 0.80%); however, this difference represented only a non-significant trend (*p* = 0.07) (Table 1 and Figure 2).

No significant differences were detected at the expanded-blastocyst stage. The proportion of morulae developing to expanded and hatched blastocysts was comparable between groups on Day 7 (*p* > 0.05) (Table 1 and Figure 2), demonstrating that PL supplementation did not significantly alter the output of expanded and hatched blastocysts.

A distinct effect of PL supplementation emerged at the hatching stage. On Day 8, the hatched-blastocyst rate was higher in the PL-group than in the C-group (*p* = 0.007) (Table 1 and Figure 2), indicating that PL supplementation specifically accelerated blastocyst hatching.

Finally, when overall efficiency was assessed as the cumulative blastocyst yield (expanded plus hatched blastocysts on Day 8) relative to the allocated COCs, the PL-group showed a numerically higher yield than the C-group (42.75 ± 4.00% vs. 28.28 ± 1.97%); this difference represented only a non-significant trend (*p* = 0.06) (Table 1).

**Figure 3:**
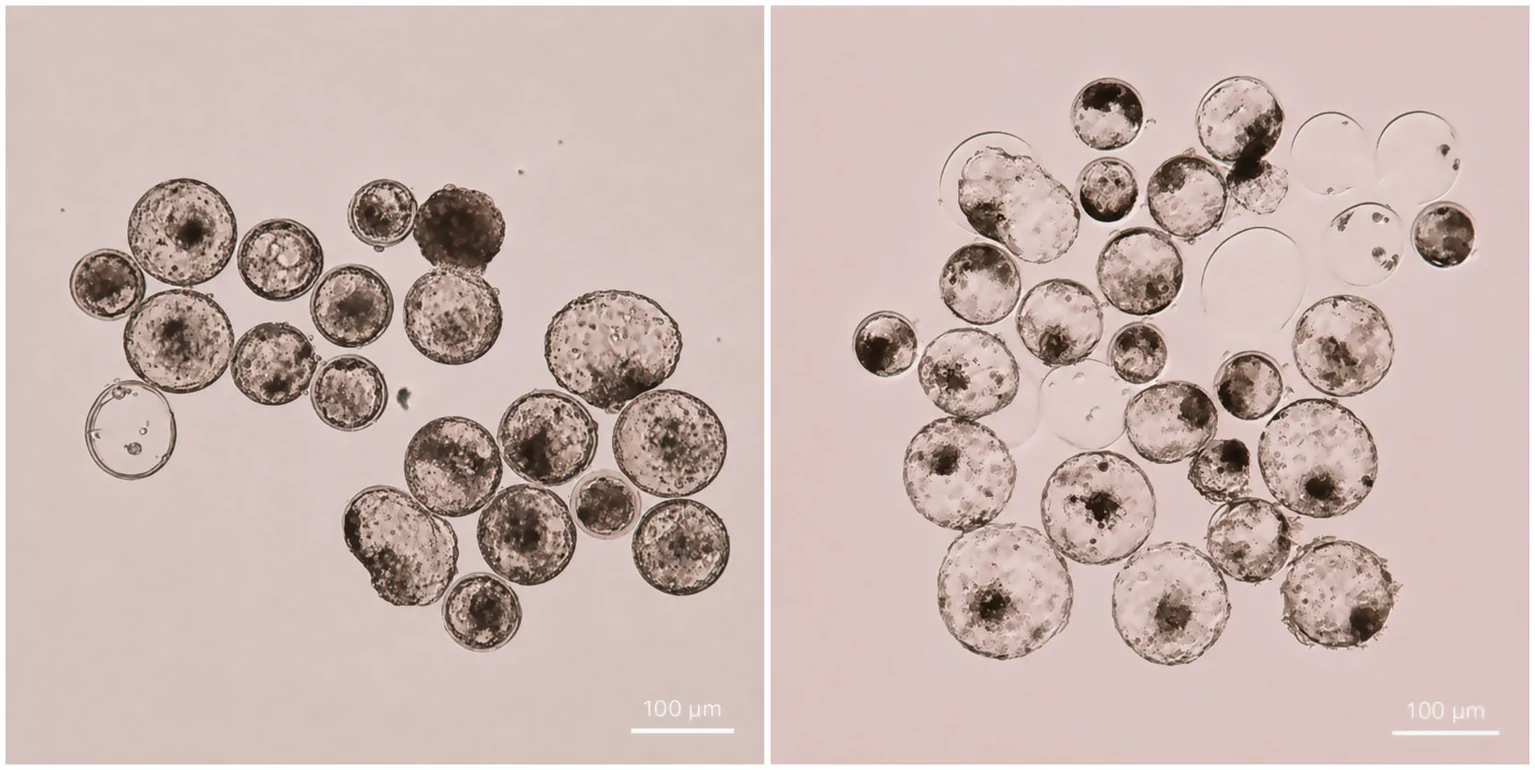
Representative stereomicroscopic captures of bovine embryos on Day 8 of IVC (5 × total magnification; scale bar = 100 μm). (Left) Control group (C-group) presenting a population predominantly composed of unhatched, expanded blastocysts classified under IETS Stage 6, Code 1/2 (Excellent to Fair) (37). (Right) Platelet Lysate (PL-group) supplemented medium showcasing a highly advanced developmental shift with a prominent proportion of fully hatched blastocysts successfully shed from the zona pellucida, classified under IETS Stage 9, Code 1 (Excellent/Good) (37), visually substantiating the accelerated hatching kinetics described in the quantitative analysis.

Taken together, these results indicate that, under the present session-level analysis (independent session as the experimental unit; *n* = 5), the principal and statistically robust effect of PL supplementation was a significant increase in the proportion of hatched blastocysts on Day 8 (Figures 2, 3).

## 4 Discussion

The use of PRP and PL has attracted considerable attention because of its potential benefits in various healing processes and *in vitro* embryo culture, as highlighted in several studies (16–18, 22, 23). Currently, there is limited research examining the specific effects of PL on *in vitro* embryo culture. It remains uncertain how PL may contribute to the enhancement of various stages in embryo development. Further research is needed to elucidate the specific mechanisms through which PL influences cellular processes, growth factors, and the overall environment that supports embryo development. Understanding these interactions could provide valuable insights into potential applications in reproductive medicine.

Given its well-known mitogenic properties and high concentration of growth factors (11, 12, 24), we hypothesized that using PL obtained from estrus heifers during *in vitro* embryo culture could provide a viable and cost-effective strategy to improve embryo development and hatching rates. These pathways are thought to be influenced by various growth factors and cytokines found in the *α*-granules of platelets. The α-granules contain growth factors such as platelet-derived growth factor, transforming growth factor-β, epidermal growth factor, vascular endothelial growth factor, fibroblast growth factors, insulin-like growth factor, and hepatocyte growth factor (25, 26). These growth factors, which are commonly associated with PRP and PL, may play a role in the regeneration of both the endometrium and ovaries (25, 26). Furthermore, some of the previously mentioned growth factors have demonstrated positive outcomes when used as additives in human IVF culture media, including increased pregnancy rates, faster development, improved quality, higher rates of blastocyst formation, increased cell numbers, earlier hatching, and better implantation rates (10, 27). Consequently, our study aimed to evaluate the IVEP potential of IVF media when supplemented with PL compared with non-supplemented media during the IVC procedures. The initial findings from our study provide strong evidence that supplementation with PL from estrus heifers significantly increases the rate of hatched blastocysts. Specifically, the results indicate a marked improvement in embryonic developmental outcomes when PL is incorporated into the culture medium.

These outcomes suggest that PL not only plays a critical role in promoting cell growth and development but could also be a valuable additive for optimizing the conditions under which embryos are cultured. By improving embryo hatching rates, PL holds promise for advancing techniques in reproductive biotechnology and enhancing success rates in assisted reproductive technologies.

In this study, the IVF media supported embryo production at rates comparable to those reported in other studies, provided that they retained the characteristics recommended by the manufacturer before use. All media exhibited consistent pH levels, color, proper viscosity, and lacked visible deposits, in alignment with the manufacturer’s recommendations before use. These findings suggest that the media’s buffering capacity likely remained stable. This assumption is supported by visual observations, which indicate that the macroscopic characteristics of the used media appear suitable for IVEP during and after the equilibration period. Similar findings were reported by Hossain et al. (2), who confirmed these results through pH evaluation of the media under three different treatment conditions. They observed that the alkalinity of the IVF media is influenced by both the duration and the volume of media tested. Notably, it is also well established that all bicarbonate-buffered media tend to become alkaline when exposed to ambient air (1, 28, 29). However, to preserve their macroscopic characteristics until use, the IVF media used in this study were protected from exposure to ambient air, light, and temperature fluctuations.

In this study, we included all COCs collected for the IVF protocols, even though lower-quality COCs are generally not recommended for IVF due to their reduced fertilization potential and overall success rates. The classification criteria for COCs can vary among laboratories. De Bem et al. (30) indicated that class III COCs, which are categorized as having poor morphological quality, performed better in terms of blastocyst development than those categorized as class II (medium quality). Interestingly, class III COCs showed similar blastocyst development rates to class I COCs, which are considered the highest quality, although there were no differences in blastocyst quality among these groups. Bilodeau-Goeseels et al. (31) further divided COCs into six classes based on features of the cumulus and ooplasm. They discovered that oocytes with fewer than five layers of cumulus cells had lower cleavage rates; however, their potential for development to the blastocyst stage was comparable to that of oocytes with more than five layers of cumulus cells. Despite these seemingly contradictory findings, most studies agree that COCs exhibiting signs of early atresia yield higher blastocyst rates compared to morphologically healthy ones (32).

Notably, the culture medium supplemented with PL derived from an estrus heifer produced developmental outcomes comparable to those reported in other studies. In the present study, the 2-cell (cleavage) rates were comparable between the two groups (70.36 ± 3.73% vs. 64.85 ± 4.15%; *p* = 0.46), consistent with the findings of Lange-Consiglio et al. (16), who reported a cleavage rate of 66.80% with no significant difference after the inclusion of platelet concentrates in bovine embryo culture. These data indicate that PL supplementation does not significantly influence initial cleavage kinetics in bovine IVEP.

The subsequent morula formation rate derived from cleaved 2-cell embryos was numerically higher in the C-group than in the PL-group (75.83 ± 3.25% vs. 66.61 ± 0.80%); however, this difference did not reach statistical significance (*p* = 0.07) and therefore represents only a non-significant trend. Should such a numerical delay in the PL cohort be confirmed in larger studies, it might reflect a transient threshold or modulatory effect of specific growth factors or cytokines within the lysate at this developmental transition, given that the optimal concentration of individual signaling molecules during early bovine development remains to be established (28).

This early numerical difference did not translate into reduced blastocyst formation. The proportion of expanded blastocysts on Day 8 derived from morulae was higher in the PL-group (50.17 ± 2.73% vs. 41.77 ± 5.05%), although this difference was not statistically significant (*p* = 0.11). Importantly, the hatched-blastocyst rate derived from morulae was higher in the PL-supplemented medium, reaching 40.33 ± 3.54% compared with 16.71 ± 1.27% in the C-group (*p* = 0.007). Consistently, the cumulative blastocyst yield from the initial pool of recovered COCs was higher in the PL-group (42.75 ± 4.00% vs. 28.28 ± 1.97%), a difference that approached but did not reach statistical significance (*p* = 0.06). The direction of these effects is in line with Ramos-Deus et al. (17), who reported increased blastocyst production (43% vs. 33%) using 5% PRP, supporting a role for platelet-derived factors in promoting blastocyst development; in the present dataset, however, the statistically robust effect was specific to hatching rather than to overall blastocyst yield.

PRP and PL contain numerous growth factors and cytokines that significantly contribute to embryonic development prior to implantation. These bioactive components contribute to the creation of an optimal environment for embryonic growth, indicating that the intricate balance of pro-inflammatory and anti-inflammatory cytokines in the immune response is essential for promoting healthy embryonic development. When this balance is maintained, cytokines may positively influence the surrounding milieu, supporting embryonic progression. Moreover, PRP and PL interaction with additional growth factors, such as vascular endothelial growth factor and transforming growth factor-beta, may further contribute to the enhancement of embryonic development by promoting cellular proliferation. This multifaceted interplay between PRP and PL components, as well as other growth factors, underscores the potential role of platelets in improving outcomes in assisted reproductive technologies (33–36).

To the best of our knowledge, this study represents the first preliminary investigation evaluating the effect of adding PL, specifically derived from blood donors in a validated state of spontaneous estrus, into culture media during bovine IVEP. While Hassaneen et al. (18) demonstrated that generic PL supports bovine embryo development *in vitro*, our approach characterizes the unique synergistic potential of combining platelet-released granular fractions with the specific hormonal and systemic metabolic background characteristic of the heifer’s estrus cycle. Because PRP and PL are typically derived from autologous or matched allogeneic blood, this bioprocess significantly mitigates risks related to cross-species immunogenicity, providing a robust, safe, and cost-effective biotechnical alternative to fetal bovine serum (FBS) for clinical and commercial embryo production.

Numerous studies have investigated the beneficial effects of PRP, particularly highlighting its role in promoting cellular proliferation and differentiation. However, a major challenge remains the lack of standardized protocols for PRP preparation, leading to a wide variability in the methodologies employed across different research settings. This lack of uniformity hampers the reproducibility of results and complicates comparisons among studies, as no consensus has been reached regarding the optimal PRP concentration for specific applications. Therefore, further research is warranted to establish standardized guidelines that could help streamline PRP and PL applications in reproductive technologies and enhance bovine IVEP outcomes.

This study is limited by the fact that the concentrations of white blood cells, red blood cells and platelets in the PRP were not assessed, which may affect the characteristics of PL. Nevertheless, a standard PRP kit sourced from human medicine was employed, a method that shows promise and could be further explored in different research contexts. To fully clarify the effects of PRP and PL in embryo culture, further large-scale randomized controlled trials in bovine are necessary.

## 5 Conclusion

Our preliminary investigation indicates that the inclusion of PL derived from an estrus heifer in the culture medium has a significant positive effect specifically on blastocyst hatching. The most robust effect was a significant increase in the proportion of hatched blastocysts on Day 8, whereas the overall (cumulative) blastocyst yield showed a positive but non-significant trend.

This finding strongly suggests that the addition of PL to the culture medium could be a highly effective strategy for improving the developmental potential of bovine embryos. By providing essential growth factors and nutrients, PL supplementation appears to facilitate the embryos’ progress to the critical hatched stage, which is essential for successful implantation and subsequent development.

Overall, these insights underscore the potential benefits of integrating appropriately preserved IVF media with PL supplementation. This combination may optimize the outcomes of bovine IVEP, leading to enhanced efficiency and success rates in embryo development. Such advancements could have significant implications for improving reproductive technologies in the cattle industry.

## Data Availability

The raw data supporting the conclusions of this article will be made available by the authors, without undue reservation.
